# Synthesis, crystal structure and Hirshfeld surface analysis of *N*-(4-fluoro­phen­yl)-*N*-isopropyl-2-(methyl­sulfon­yl)acetamide

**DOI:** 10.1107/S2056989023003675

**Published:** 2023-04-28

**Authors:** Doreswamy Geetha, Haleyur G. Anil Kumar, Thaluru M. Mohan Kumar, Gejjalagere R. Srinivasa, Yeriyur B. Basavaraju, Hemmige S. Yathirajan, Sean Parkin

**Affiliations:** a Department of Studies in Chemistry, University of Mysore, Manasagangotri, Mysuru-570 006, India; b Department of Science and Humanities, PES University, BSK III Stage, Bengaluru-560 085, India; c Department of Chemistry, Amrita School of Engineering, Amrita Vishwa Vidyapeetham, Bengaluru-560 035, India; d Honeychem Pharma Research Pvt. Ltd., Peenya Industrial Area, Bengaluru-560 058, India; e Department of Chemistry, University of Kentucky, Lexington, KY, 40506-0055, USA; University of Aberdeen, United Kingdom

**Keywords:** *N*-(substituted phen­yl)acetamide, flufenacet metabolite, crystal structure, Hirshfeld surface analysis

## Abstract

The synthesis and crystal structure of the title compound, C_12_H_16_FNO_3_S, which is related to the herbicide flufenacet, are presented. The dihedral angle between the amide group and the fluorinated benzene ring is 87.30 (5)° and the N—C—C—S torsion angle defining the orientation of the methyl­sulfonyl substituent relative to the amide group is 106.91 (11)°. In the crystal, inversion-related mol­ecules form dimers as a result of pairwise C—H⋯O hydrogen bonds, which appear to be reinforced by short O⋯π contacts [O⋯*Cg* = 3.0643 (11) Å]. A Hirshfeld surface analysis was used to qu­antify the various types of inter­molecular contacts, which are dominated by H atoms.

## Abstract 2

In the title compound, which is related to the herbicide flufenacet, the amide group and fluoro­benzene ring are almost perpendicular. A short O⋯π contact is observed in the crystal.

## 1. Chemical context

*N*-(Substituted phen­yl)acetamides have a variety of biological activities. For example, substituted phenyl­acetamides and their use as protease inhibitors was reported by Kreutter *et al.* (2009 ▸) and a description of the syntheses and anti­oxidant studies of *N*-substituted benz­yl/phenyl-2-[3,4-dimethyl-5,5-dioxido­pyrazolo­[4,3-*c*][1,2]benzo­thia­zin-2(4*H*)-yl]acetamides was given by Ahmad *et al.* (2013 ▸). The syntheses and biological evaluation of *N* ^4^-substituted sulfonamide–acetamide derivatives as di­hydro­folate reductase (DHFR) inhibitors was reported by Hussein *et al.* (2019 ▸) and the synthesis of *N*-(substituted phen­yl)-*N*-(substituted)acetamide derivatives as potent analgesic agents was described by Verma *et al.* (2020 ▸). Lastly, the evaluation of new 2-hy­droxy-*N*-(4-oxo-2-substituted phenyl-1,3-thia­zolidin-3-yl)-2-phenyl­acetamide derivatives as potential anti­mycobacterial agents was reported by Güzel-Akdemir *et al.* (2020 ▸). 
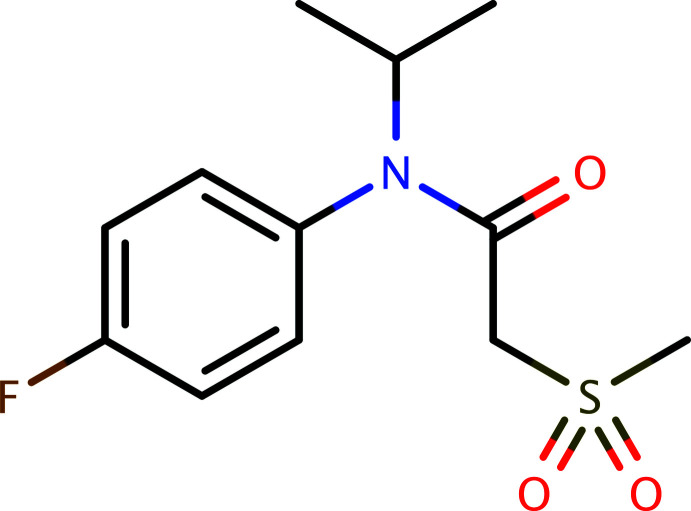


**Figure 1:**
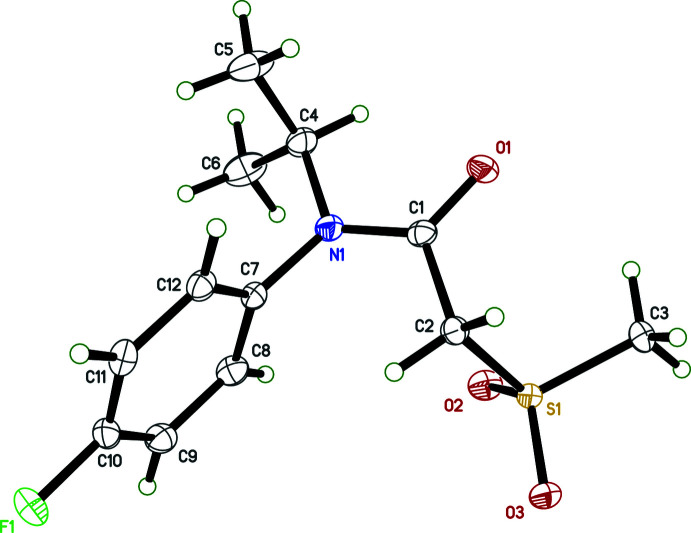
The mol­ecular structure of **I** showing 50% displacement ellipsoids.

Flufenacet (C_14_H_13_F_4_N_3_O_2_S), systematic name *N*-(4-fluoro­phen­yl)-*N*-propan-2-yl-2-{[5-(tri­fluoro­meth­yl)-1,3,4-thia­diazol-2-yl]­oxy}acetamide, is an herbi­cide, xenobiotic and environmental contaminant (Rouchaud *et al.*, 2001 ▸; Zimmerman *et al.*, 2002 ▸). This paper reports the synthesis, crystal structure and a Hirshfeld surface analysis of the related title compound, C_12_H_16_FNO_3_S (**I**) (Fig. 1 ▸).

## 2. Structural commentary

In the crystal structure of **I**, the nitro­gen atom of the amide group is close to planar, the sum of bond angles about N1 being 358.92 (19)°, which places N1 0.0862 (14) Å from the plane passing through C1, C4, and C7. The amide group is also almost planar, having an r.m.s. deviation from the mean plane of N1, C1, O1, C2 of 0.0095 Å [maximum = 0.0165 (11) Å for C1], and is almost perpendicular to the fluoro­benzene ring (C7–C12), subtending a dihedral angle of 87.30 (5)°. The overall conformation of the mol­ecule is defined by the torsion angles C7—N1—C1—C2 [14.68 (17)°], N1—C1—C2—S1 [106.91 (11)°], C1—C2—S1—C3 [74.53 (10)°] and by the orientation of the ^i^propyl group, *e.g.*, C1—N1—C4—C6 [139.85 (13)°]. Otherwise, all bond lengths and angles lie within the expected ranges.

## 3. Supra­molecular features

**Figure 2:**
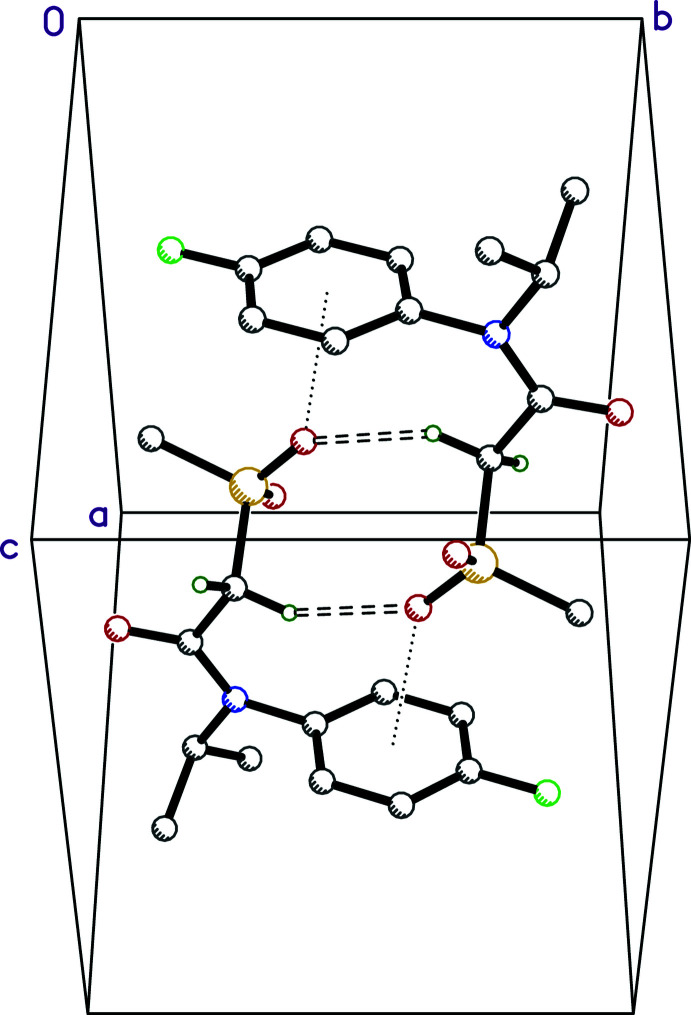
A partial packing plot of **I**, showing inversion dimers resulting from pairs of C—H⋯O weak hydrogen bonds, augmented by O⋯*Cg*(ring) contacts. Hydrogen atoms not involved in the hydrogen bonds are omitted.

**Figure 3:**
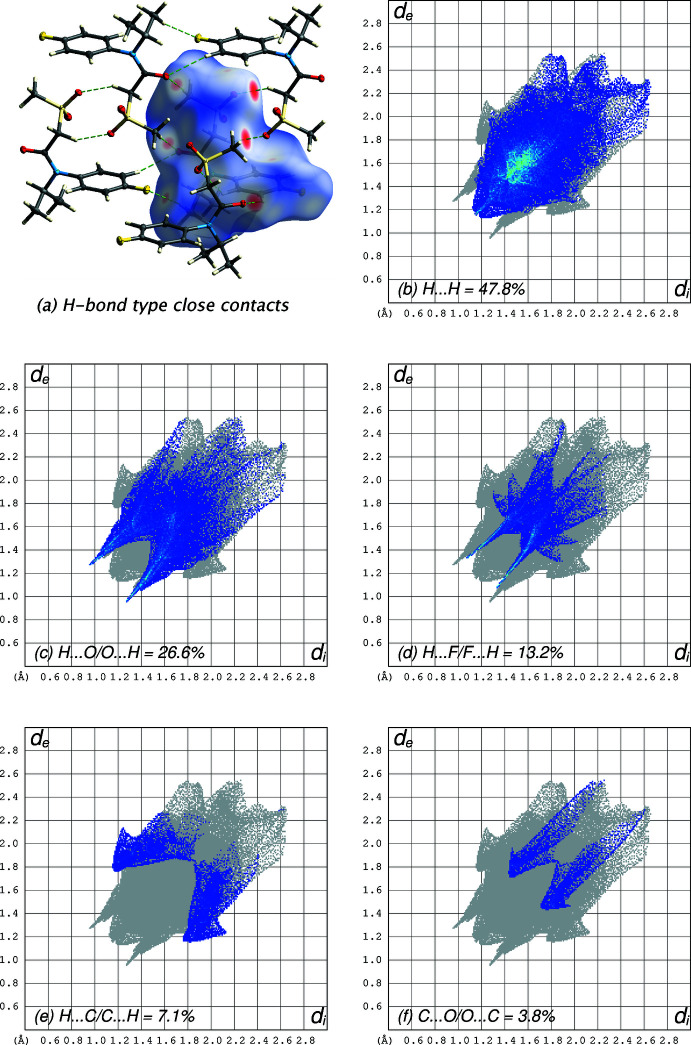
(*a*) The Hirshfeld surface of **I** expressed over *d_norm_*, with C—H⋯O and C—H⋯F inter­actions drawn as dashed lines and as dark- and light-red regions on the Hirshfeld surface, respectively; (*b*) fingerprint plot of H⋯H contacts; (*c*) H⋯O/O⋯H contacts; (*d*) H⋯F/F⋯H contacts; (*e*) H⋯C/C⋯H contacts; (*f*) C⋯O/O⋯C contacts.

There are no strong hydrogen bonds in the crystal structure of **I** (Fig. 2 ▸), but there are a number of weaker C—H⋯O and C—H⋯F inter­actions, which are qu­anti­fied in Table 1 ▸. The most prominent supra­molecular constructs are dimers in which inversion-related mol­ecules are linked by C2—H2*A*⋯O3^i^ and C2^i^—H2*A* ^i^⋯O3 hydrogen bonds [symmetry code: (i) 1 – *x*, 1 – *y*, 1 – *z*]. These dimers also feature close contacts between the sulfone O3 atom and the inversion-related benzene ring to give an O3⋯*Cg*(C7–C12)^i^ distance of 3.0643 (11) Å (*e.g.* Gung *et al.*, 2008 ▸ and see also Section 4: *Database survey*). The other weak C—H⋯O inter­actions involve inversion, translation, and *c*-glide related mol­ecules (Table 1 ▸, Fig. 3 ▸ *a*). A Hirshfeld surface analysis using *CrystalExplorer* (Spackman *et al.*, 2021 ▸) shows that almost all atom–atom contacts involve the hydrogen atoms (Fig. 3 ▸ *b*–*f*).

## 4. Database survey

A search of the Cambridge Structural Database (CSD, v5.43 with updates through November 2022; Groom *et al.*, 2016 ▸) for a mol­ecular fragment consisting of *N*-phenyl­acetamide with ‘any non-H group’ attached at the nitro­gen atom, the 4-position of the benzene ring, and replacing one hydrogen of the methyl group, yielded 259 hits. A similar fragment, but with ‘any halogen’ at the 4-position on the ring, gave 92 hits. With the halogen restricted to fluorine, twelve hits were returned, and with an isopropyl group attached to the nitro­gen atom, only one match was found: CSD refcode QEMHOG (Gao & Ng, 2006 ▸): this structure has a 1,3-benzo­thia­zol-2-yl-oxy group attached to the methyl­ene carbon atom of the search fragment.

A search of the CSD for non-bonded close contacts (up to 3.1Å) between S=O oxygen atoms and a benzene-ring centroid (with ‘any substituent’) returned 154 hits, none of which have much else in common with **I**. A crystallographic and computational study of inter­actions between oxygen lone pairs and aromatic rings (albeit involving carbon-bound oxygen atoms) was presented by Gung *et al.* (2008 ▸).

There are several other related structures in the CSD, namely: thia­mphenicol, d-threo-2,2-di­chloro-*N*-{2-hy­droxy-1-(hy­droxy­meth­yl)-2-[4-(methyl­sulfon­yl)phen­yl]eth­yl}acet­a­­mide (CABCIR01; Ghosh *et al.*, 1987 ▸), 2,2-di­chloro-*N*-{[1-(fluoro­meth­yl)-2-hy­droxy-2-[4- (methyl­sulfon­yl)phen­yl]eth­yl}acetamide (GAWNIC; Cheng *et al.*, 2005 ▸), *N*-(2,6-di­methyl­phen­yl)-2-(2-{3-[4-(methyl­sulfon­yl)phen­yl]-1,2,4-oxa­diazol-5-yl}phen­oxy)acetamide (AFIFIF; Wang *et al.*, 2007 ▸), *N*-(4-chloro-2-nitro­phen­yl)-*N*-(methyl­sulfon­yl)acetamide (WOGWEV; Zia-ur-Rehman *et al.*, 2008 ▸), *N*-(4-meth­oxy-2-nitro-phen­yl)-*N*-(methyl­sulfon­yl)acetamide (QOTNAP; Zia-ur-Rehman *et al.*, 2009 ▸), 2-chloro-*N*-(4-chloro-2-(2-chloro­benzo­yl)phen­yl)acetamide (DUPLUW; Dutkiewicz *et al.*, 2010 ▸), 2-chloro-*N*-[2-(2-fluoro­benzo­yl)-4-nitro­phen­yl]-*N*-methyl­acetamide (EXIVEN; Siddaraju *et al.*, 2011 ▸), 2-phenyl-*N*-(pyrazin-2-yl)acetamide (ROJNAH; Nayak *et al.*, 2014 ▸) and 2-(perfluoro­phen­yl)acetamide (LAMRAW; Novikov *et al.*, 2022 ▸).

## 5. Synthesis, crystallization and spectroscopic details

In a 250 ml flask (with a nitro­gen inlet and a septum) was placed 5 g of 4-fluoro-*N*-iso­propyl­benzenamine dissolved in 50 ml of aceto­nitrile. After cooling to 273 K, 6.7 g of tri­ethyl­amine and 4.11 g of 2-(methyl­thio)­acetyl chloride were added. The mixture was stirred at room temperature for 5 h. After this, 100 ml of water were added and the mixture was extracted three times, each with 100 ml of methyl tert-butyl ether (MTBE). The combined organic phases were dried over MgSO_4_ and the solvent was evaporated under reduced pressure. The crude product, *N*-(4-fluoro­phen­yl)-*N*-isopropyl-2-(methyl­thio)­acetamide, was used for the next stage with purification (7.5 g).

**Figure 4:**
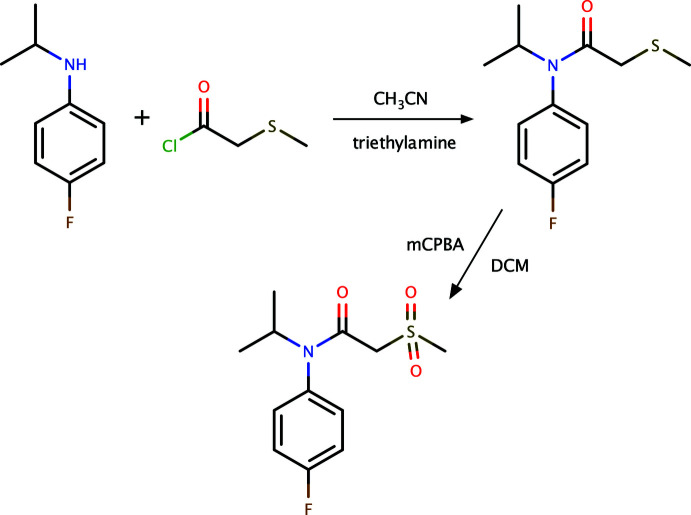
A reaction scheme for the synthesis of **I**. DCM is di­chloro­methane, mCPBA is *meta*-chloro­perbenzoic acid.

To a 250 ml round-bottomed flask (with a nitro­gen inlet and a septum) was added 7.5 g of *N*-(4-fluoro­phen­yl)-N-isopropyl-2-(methyl­thio)­acetamide dissolved in 150 ml of di­chloro­methane. After cooling to 263–273 K, 13.37 g of *meta*-chloro­perbenzoic acid in 100 ml di­chloro­methane was added slowly at the same temperature. The mixture was stirred at room temperature for 5 h. After this, 200 ml of water were added and the organic layer was separated, and washed with 100 ml of 10% sodium bicarbonate twice. The organic phases were dried over MgSO_4_ and the solvent was evaporated under reduced pressure. The crude product was purified by chromatography over SiO_2_ (hexa­ne:ethyl acetate 9:1 *v*/*v*). The title compound was recrystallized from diethyl ether solution in the form of colorless plates. The overall reaction scheme is shown in Fig. 4 ▸.

^1^H NMR: CDCl_3_ (400 MHz, δ ppm): 1.097–1.08 [6H, *d*, (CH_3_)_2_]; 3.198 (3H, *s*, –CH_3_); 3.664 (2H, *s*, CH_2_); 5.006–4.938 (1H, *m*, –CH); 7.273–7.132 (4H, *m*, ar H). MS *m*/*z*: 273.45 (*M*)^+^.

## 6. Refinement

Crystal data, data collection and structure refinement details are summarized in Table 2 ▸. Hydrogen atoms were found in difference-Fourier maps, but subsequently included in the refinement using riding models, with constrained C—H distances set to 0.95 Å (C*sp* ^2^H), 0.98 Å (*R*CH_3_), 0.99 Å (*R*_2_CH_2_) and 1.00 Å (*R*_3_CH). *U*_iso_(H) parameters were set to values of either 1.2*U*_eq_ or 1.5*U*_eq_ (*R*CH_3_ only) of the attached atom.

## Supplementary Material

Crystal structure: contains datablock(s) I, global. DOI: 10.1107/S2056989023003675/hb8062sup1.cif

Structure factors: contains datablock(s) I. DOI: 10.1107/S2056989023003675/hb8062Isup2.hkl

Supporting information file. DOI: 10.1107/S2056989023003675/hb8062Isup3.cml

CCDC reference: 2258160

Additional supporting information:  crystallographic information; 3D view; checkCIF report

## Figures and Tables

**Table 1:** Weak hydrogen bonds and other short inter­molecular contacts (Å, °)

*D*—H⋯*A*	*D*—H	H⋯*A*	*D*⋯*A*	*D*—H⋯*A*
C2—H2*A*⋯O3^i^	0.99	2.31	3.2025 (16)	150
C3—H3*B*⋯O1^ii^	0.98	2.55	3.5196 (17)	170
C5—H5*A*⋯F1^iii^	0.98	2.50	3.4295 (18)	158
C11—H11⋯O1^iv^	0.95	2.39	3.1851 (16)	141
C12—H12⋯O2^iv^	0.95	2.56	3.2955 (16)	134
O3⋯*Cg*(C7–C12)^i^			3.0643 (11)	

Symmetry codes: (i) −*x* + 1, −*y* + 1, −*z* + 1; (ii) −*x* + 1, −*y*, −*z* + 1; (iii) *x*, *y* − 1, *z*; (iv) *x*, −*y* + 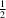 , *z* + 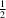 .

**Table 2:** Experimental details

Crystal data	
Chemical formula	C_12_H_16_FNO_3_S
*M*_r_	273.32
Crystal system, space group	Monoclinic, *P*2_1_/*c*
Temperature (K)	90
*a*, *b*, *c* (Å)	12.9530 (3), 8.7657 (2), 11.7723 (3)
β (°)	100.457 (1)
*V* (Å^3^)	1314.45 (5)
*Z*	4
Radiation type	Mo *K*α
μ (mm^−1^)	0.26
Crystal size (mm)	0.32 × 0.31 × 0.09

Data collection	
Diffractometer	Bruker D8 Venture dual source
Absorption correction	Multi-scan (*SADABS*; Krause *et al.*, 2015 ▸)
*T*_min_, *T*_max_	0.833, 0.971
No. of measured, independent and observed [*I* > 2σ(*I*)] reflections	22801, 3010, 2678
*R*_int_	0.030
(sin θ/λ)_max_ (Å^−1^)	0.650

Refinement	
*R*[*F* ^2^ > 2σ(*F* ^2^)], *wR*(*F* ^2^), *S*	0.031, 0.079, 1.05
No. of reflections	3010
No. of parameters	166
H-atom treatment	H-atom parameters constrained
Δρ_max_, Δρ_min_ (e Å^−3^)	0.36, −0.37

Computer programs: *APEX3* (Bruker, 2016 ▸), *SHELXT* (Sheldrick, 2015*a* ▸), *SHELXL2019/2* (Sheldrick, 2015*b* ▸), *XP* in *SHELXTL* (Sheldrick, 2008 ▸), *CrystalExplorer* (Spackman *et al.*, 2021 ▸), *SHELXTL* (Sheldrick, 2008 ▸), and *publCIF* (Westrip, 2010 ▸).

## supplementary crystallographic information

### Crystal data

**Table:**

C_12_H_16_FNO_3_S	*F*(000) = 576
*M**_r_* = 273.32	*D*_x_ = 1.381 Mg m^−^^3^
Monoclinic, *P*2_1_/*c*	Mo *K*α radiation, λ = 0.71073 Å
*a* = 12.9530 (3) Å	Cell parameters from 9889 reflections
*b* = 8.7657 (2) Å	θ = 2.8–27.5°
*c* = 11.7723 (3) Å	µ = 0.26 mm^−^^1^
β = 100.457 (1)°	*T* = 90 K
*V* = 1314.45 (5) Å^3^	Rounded plate, colourless
*Z* = 4	0.32 × 0.31 × 0.09 mm

### Data collection

**Table:**

Bruker D8 Venture dual source diffractometer	3010 independent reflections
Radiation source: microsource	2678 reflections with *I* > 2σ(*I*)
Detector resolution: 7.41 pixels mm^-1^	*R*_int_ = 0.030
φ and ω scans	θ_max_ = 27.5°, θ_min_ = 2.8°
Absorption correction: multi-scan (*SADABS*; Krause *et al.*, 2015)	*h* = −16→16
*T*_min_ = 0.833, *T*_max_ = 0.971	*k* = −11→11
22801 measured reflections	*l* = −15→15

### Refinement

**Table:**

Refinement on *F*^2^	Primary atom site location: structure-invariant direct methods
Least-squares matrix: full	Secondary atom site location: difference Fourier map
*R*[*F*^2^ > 2σ(*F*^2^)] = 0.031	Hydrogen site location: difference Fourier map
*wR*(*F*^2^) = 0.079	H-atom parameters constrained
*S* = 1.05	*w* = 1/[σ^2^(*F*_o_^2^) + (0.0327*P*)^2^ + 0.7883*P*] where *P* = (*F*_o_^2^ + 2*F*_c_^2^)/3
3010 reflections	(Δ/σ)_max_ = 0.001
166 parameters	Δρ_max_ = 0.36 e Å^−^^3^
0 restraints	Δρ_min_ = −0.37 e Å^−^^3^

### Special details

**Table:**

Experimental. The crystal was mounted using polyisobutene oil on the tip of a fine glass fibre, which was fastened in a copper mounting pin with electrical solder. It was placed directly into the cold gas stream of a liquid-nitrogen based cryostat (Hope, 1994; Parkin & Hope, 1998). Diffraction data were collected with the crystal at 90K, which is standard practice in this laboratory for the majority of flash-cooled crystals.
Geometry. All esds (except the esd in the dihedral angle between two l.s. planes) are estimated using the full covariance matrix. The cell esds are taken into account individually in the estimation of esds in distances, angles and torsion angles; correlations between esds in cell parameters are only used when they are defined by crystal symmetry. An approximate (isotropic) treatment of cell esds is used for estimating esds involving l.s. planes.
Refinement. Refinement progress was checked using *Platon* (Spek, 2020) and by an *R*-tensor (Parkin, 2000). The final model was further checked with the IUCr utility *checkCIF*.

### Fractional atomic coordinates and isotropic or equivalent isotropic displacement parameters (Å^2^)

**Table:**

	*x*	*y*	*z*	*U*_iso_*/*U*_eq_	
S1	0.54145 (2)	0.29322 (3)	0.38570 (3)	0.01394 (9)	
F1	0.77308 (7)	0.83954 (10)	0.77262 (8)	0.0265 (2)	
C1	0.70051 (10)	0.18349 (15)	0.54790 (11)	0.0153 (3)	
N1	0.78957 (9)	0.26470 (13)	0.58233 (10)	0.0169 (2)	
O1	0.69978 (7)	0.04745 (11)	0.52217 (8)	0.0193 (2)	
C2	0.59691 (10)	0.26923 (15)	0.53498 (11)	0.0154 (3)	
H2A	0.608749	0.370378	0.572495	0.018*	
H2AB	0.547429	0.211700	0.573787	0.018*	
O2	0.62454 (7)	0.33486 (11)	0.32511 (8)	0.0208 (2)	
O3	0.45431 (7)	0.39662 (11)	0.38023 (8)	0.0201 (2)	
C3	0.49181 (11)	0.11358 (15)	0.33711 (12)	0.0208 (3)	
H3A	0.459299	0.120203	0.255330	0.031*	
H3B	0.439182	0.081074	0.382298	0.031*	
H3C	0.549247	0.039248	0.346600	0.031*	
C9	0.7902 (1)	0.68848 (16)	0.61179 (12)	0.0191 (3)	
H9	0.794529	0.777562	0.566931	0.023*	
C8	0.79425 (10)	0.54409 (16)	0.56408 (12)	0.0188 (3)	
H8	0.801145	0.533426	0.485496	0.023*	
C7	0.7882 (1)	0.41507 (15)	0.63150 (11)	0.0154 (3)	
C6	0.97208 (12)	0.2845 (2)	0.54665 (14)	0.0295 (3)	
H6A	1.036051	0.225348	0.545252	0.044*	
H6B	0.988878	0.372611	0.597886	0.044*	
H6C	0.943028	0.319875	0.468411	0.044*	
C5	0.93224 (12)	0.1303 (2)	0.71320 (14)	0.0309 (4)	
H5A	0.877506	0.070798	0.740427	0.046*	
H5B	0.950692	0.218725	0.763798	0.046*	
H5C	0.994531	0.066468	0.714444	0.046*	
C4	0.89195 (10)	0.18467 (16)	0.59065 (13)	0.0216 (3)	
H4	0.880049	0.092462	0.539939	0.026*	
C10	0.77975 (10)	0.69895 (15)	0.72594 (12)	0.0180 (3)	
C11	0.77354 (10)	0.57396 (16)	0.79470 (11)	0.0174 (3)	
H11	0.766284	0.585728	0.873082	0.021*	
C12	0.77814 (10)	0.42986 (15)	0.74673 (11)	0.0162 (3)	
H12	0.774416	0.341486	0.792531	0.019*	

### Atomic displacement parameters (Å^2^)

**Table:**

	*U*^11^	*U*^22^	*U*^33^	*U*^12^	*U*^13^	*U*^23^
S1	0.01361 (16)	0.01232 (15)	0.01522 (16)	0.00096 (11)	0.00086 (11)	−0.00046 (11)
F1	0.0312 (5)	0.0167 (4)	0.0308 (5)	−0.0009 (3)	0.0033 (4)	−0.0072 (3)
C1	0.0176 (6)	0.0165 (6)	0.0114 (6)	0.0028 (5)	0.0012 (5)	0.0006 (5)
N1	0.0158 (5)	0.0159 (5)	0.0179 (5)	0.0033 (4)	0.0001 (4)	−0.0022 (4)
O1	0.0224 (5)	0.0142 (5)	0.0202 (5)	0.0033 (4)	0.0015 (4)	−0.0020 (4)
C2	0.0164 (6)	0.0151 (6)	0.0142 (6)	0.0012 (5)	0.0016 (5)	−0.0018 (5)
O2	0.0210 (5)	0.0217 (5)	0.0208 (5)	0.0013 (4)	0.0070 (4)	0.0049 (4)
O3	0.0170 (5)	0.0180 (5)	0.0237 (5)	0.0049 (4)	−0.0007 (4)	−0.0018 (4)
C3	0.0208 (7)	0.0155 (6)	0.0243 (7)	−0.0016 (5)	−0.0004 (5)	−0.0058 (5)
C9	0.0184 (6)	0.0176 (6)	0.0212 (7)	−0.0010 (5)	0.0030 (5)	0.0043 (5)
C8	0.0194 (6)	0.0220 (7)	0.0152 (6)	0.0020 (5)	0.0038 (5)	0.0017 (5)
C7	0.0124 (6)	0.0158 (6)	0.0171 (6)	0.0004 (5)	0.0002 (5)	−0.0017 (5)
C6	0.0227 (7)	0.0418 (9)	0.0258 (8)	0.0094 (7)	0.0093 (6)	0.0081 (7)
C5	0.0184 (7)	0.0345 (9)	0.0389 (9)	0.0043 (6)	0.0030 (6)	0.0195 (7)
C4	0.0155 (6)	0.0222 (7)	0.0258 (7)	0.0052 (5)	0.0003 (5)	−0.0036 (6)
C10	0.0147 (6)	0.0158 (6)	0.0227 (7)	−0.0007 (5)	0.0010 (5)	−0.0035 (5)
C11	0.0148 (6)	0.0225 (7)	0.0148 (6)	−0.0027 (5)	0.0021 (5)	−0.0026 (5)
C12	0.0140 (6)	0.0181 (6)	0.0160 (6)	−0.0021 (5)	0.0010 (5)	0.0025 (5)

### Geometric parameters (Å, º)

**Table:**

S1—O3	1.4399 (9)	C9—H9	0.9500
S1—O2	1.4419 (10)	C8—C7	1.3920 (18)
S1—C3	1.7570 (13)	C8—H8	0.9500
S1—C2	1.7862 (13)	C7—C12	1.3922 (18)
F1—C10	1.3586 (15)	C6—C4	1.519 (2)
C1—O1	1.2300 (16)	C6—H6A	0.9800
C1—N1	1.3535 (17)	C6—H6B	0.9800
C1—C2	1.5213 (17)	C6—H6C	0.9800
N1—C7	1.4411 (16)	C5—C4	1.519 (2)
N1—C4	1.4876 (16)	C5—H5A	0.9800
C2—H2A	0.9900	C5—H5B	0.9800
C2—H2AB	0.9900	C5—H5C	0.9800
C3—H3A	0.9800	C4—H4	1.0000
C3—H3B	0.9800	C10—C11	1.3732 (19)
C3—H3C	0.9800	C11—C12	1.3892 (19)
C9—C10	1.378 (2)	C11—H11	0.9500
C9—C8	1.3896 (19)	C12—H12	0.9500
			
O3—S1—O2	117.90 (6)	C8—C7—C12	120.32 (12)
O3—S1—C3	108.18 (6)	C8—C7—N1	120.53 (12)
O2—S1—C3	109.13 (7)	C12—C7—N1	119.13 (12)
O3—S1—C2	106.87 (6)	C4—C6—H6A	109.5
O2—S1—C2	108.27 (6)	C4—C6—H6B	109.5
C3—S1—C2	105.84 (7)	H6A—C6—H6B	109.5
O1—C1—N1	123.48 (12)	C4—C6—H6C	109.5
O1—C1—C2	119.15 (12)	H6A—C6—H6C	109.5
N1—C1—C2	117.29 (11)	H6B—C6—H6C	109.5
C1—N1—C7	121.96 (11)	C4—C5—H5A	109.5
C1—N1—C4	118.22 (11)	C4—C5—H5B	109.5
C7—N1—C4	118.74 (11)	H5A—C5—H5B	109.5
C1—C2—S1	110.27 (9)	C4—C5—H5C	109.5
C1—C2—H2A	109.6	H5A—C5—H5C	109.5
S1—C2—H2A	109.6	H5B—C5—H5C	109.5
C1—C2—H2AB	109.6	N1—C4—C6	111.23 (12)
S1—C2—H2AB	109.6	N1—C4—C5	111.03 (12)
H2A—C2—H2AB	108.1	C6—C4—C5	111.49 (12)
S1—C3—H3A	109.5	N1—C4—H4	107.6
S1—C3—H3B	109.5	C6—C4—H4	107.6
H3A—C3—H3B	109.5	C5—C4—H4	107.6
S1—C3—H3C	109.5	F1—C10—C11	118.07 (12)
H3A—C3—H3C	109.5	F1—C10—C9	118.66 (12)
H3B—C3—H3C	109.5	C11—C10—C9	123.26 (12)
C10—C9—C8	118.19 (12)	C10—C11—C12	118.32 (12)
C10—C9—H9	120.9	C10—C11—H11	120.8
C8—C9—H9	120.9	C12—C11—H11	120.8
C9—C8—C7	119.96 (12)	C11—C12—C7	119.94 (12)
C9—C8—H8	120.0	C11—C12—H12	120.0
C7—C8—H8	120.0	C7—C12—H12	120.0
			
O1—C1—N1—C7	−168.59 (12)	C1—N1—C7—C12	78.93 (16)
C2—C1—N1—C7	14.68 (17)	C4—N1—C7—C12	−88.93 (15)
O1—C1—N1—C4	−0.67 (19)	C1—N1—C4—C6	139.85 (13)
C2—C1—N1—C4	−177.40 (11)	C7—N1—C4—C6	−51.84 (16)
O1—C1—C2—S1	−69.97 (14)	C1—N1—C4—C5	−95.38 (15)
N1—C1—C2—S1	106.91 (11)	C7—N1—C4—C5	72.93 (16)
O3—S1—C2—C1	−170.32 (9)	C8—C9—C10—F1	−178.38 (11)
O2—S1—C2—C1	−42.36 (11)	C8—C9—C10—C11	0.2 (2)
C3—S1—C2—C1	74.53 (10)	F1—C10—C11—C12	178.69 (11)
C10—C9—C8—C7	−0.3 (2)	C9—C10—C11—C12	0.1 (2)
C9—C8—C7—C12	−0.03 (19)	C10—C11—C12—C7	−0.36 (19)
C9—C8—C7—N1	178.39 (12)	C8—C7—C12—C11	0.34 (19)
C1—N1—C7—C8	−99.51 (15)	N1—C7—C12—C11	−178.10 (11)
C4—N1—C7—C8	92.63 (15)		

### Hydrogen-bond geometry (Å, º)

**Table:**

*D*—H···*A*	*D*—H	H···*A*	*D*···*A*	*D*—H···*A*
C2—H2*A*···O3^i^	0.99	2.31	3.2025 (16)	150
C3—H3*B*···O1^ii^	0.98	2.55	3.5196 (17)	170
C5—H5*A*···F1^iii^	0.98	2.50	3.4295 (18)	158
C11—H11···O1^iv^	0.95	2.39	3.1851 (16)	141
C12—H12···O2^iv^	0.95	2.56	3.2955 (16)	134

Symmetry codes: (i) −*x*+1, −*y*+1, −*z*+1; (ii) −*x*+1, −*y*, −*z*+1; (iii) *x*, *y*−1, *z*; (iv) *x*, −*y*+1/2, *z*+1/2.
